# An Enigmatic Wild Passerine Mortality Event in the Eastern United States

**DOI:** 10.3390/vetsci12010048

**Published:** 2025-01-11

**Authors:** Sabrina S. Greening, Julie C. Ellis, Nicole L. Lewis, David B. Needle, Cristina M. Tato, Susan Knowles, Valerie Shearn-Bochsler, Jaimie L. Miller, Daniel A Grear, Jeffrey M. Lorch, David S. Blehert, Caitlin Burrell, Lisa A. Murphy, Erica A. Miller, C. Brandon Ogbunugafor, Andrea J. Ayala, W. Kelley Thomas, Joseph L. Sevigny, Lawrence M. Gordon, Tessa Baillargeon, Lusajo Mwakibete, Megan Kirchgessner, Christine L. Casey, Ethan Barton, Michael J. Yabsley, Eman Anis, Roderick B. Gagne, Patrice Klein, Cindy Driscoll, Chelsea A. Sykes, Robert H. Poppenga, Nicole M. Nemeth

**Affiliations:** 1 Department of Pathobiology, Wildlife Futures Program, University of Pennsylvania School of Veterinary Medicine, New Bolton Center, Kennett Square, PA 19348, USA; millerer@upenn.edu (E.A.M.); murphylp@vet.upenn.edu (L.A.M.); 2 Office of Fish and Wildlife Health and Forensics, New Jersey DEP Fish and Wildlife, Hampton, NJ 08827, USA; 3 New Hampshire Veterinary Diagnostic Lab, University of New Hampshire, Durham, NH 03824, USAdavid.needle@unh.edu (D.B.N.); 4 Chan Zuckerberg Biohub, San Francisco, CA 94158, USA; 5 U.S. Geological Survey, National Wildlife Health Center, Madison, WI 53711, USA; 6 Department of Pathobiological Sciences, School of Veterinary Medicine, University of Wisconsin-Madison, Madison, WI 53706, USA; 7 Southeastern Cooperative Wildlife Disease Study, College of Veterinary Medicine, University of Georgia, Athens, GA 30602, USAmyabsley@uga.edu (M.J.Y.);; 8 Department of Pathology, College of Veterinary Medicine, University of Georgia, Athens, GA 30602, USA; 9 Zoo and Exotic Animal Pathology Service, Infectious Diseases Laboratory, University of Georgia, Athens, GA 30602, USA; 10 Small Animal Medicine and Surgery, University of Georgia College of Veterinary Medicine, Athens, GA 30602, USA; 11 California Animal Health and Food Safety Laboratories, School of Veterinary Medicine, University of California, Davis, CA 95616, USA; 12 Department of Ecology and Evolutionary Biology, Yale University, New Haven, CT 06511, USA; 13 Department of Natural Resources and the Environment, University of New Hampshire, Durham, NH 03824, USA; 14 Hubbard Center for Genome Studies, University of New Hampshire, Durham, NH 03824, USA; lawrence.gordon@unh.edu (L.M.G.); joseph.sevigny@unh.edu (J.L.S.); kelley.thomas@unh.edu (W.K.T.); 15 Virginia Department of Wildlife Resources, Blacksburg, VA 24060, USA; 16 U.S. Fish and Wildlife Service, Wildlife Health Office, Blacksburg, VA 24060, USA; 17 Kentucky Department of Fish and Wildlife Resources, Frankfort, KY 40601, USA; 18 West Virginia Division of Natural Resources, Elkins, WV 26241, USA; 19 Center for Ecology of Infectious Diseases, University of Georgia, Athens, GA 30602, USA; 20 Warnell School of Forestry and Natural Resources, University of Georgia, Athens, GA 30602, USA; 21 Department of Pathobiology, PADLS New Bolton Center, University of Pennsylvania School of Veterinary Medicine, New Bolton Center, Kennett Square, PA 19348, USA; 22 U.S. Department of Agriculture, Forest Service, Research and Development, Landscape and Ecosystem Services Research, Washington, DC 20250, USA; patrice.n.klein@usda.gov; 23 Maryland Department of Natural Resources, Oxford, MD 21654, USA

**Keywords:** songbird, passerines, mass mortality, wildlife investigation, conjunctivitis, diagnostic evaluation

## Abstract

The ability to rapidly respond to wildlife health events is essential. However, such events are often unpredictable, especially with anthropogenic disturbances and climate-related environmental changes driving unforeseen threats. Many events also are short-lived and go undocumented, making it difficult to draw on lessons learned from past investigations. We report on the response to a mortality event observed predominantly in wild passerines in the eastern United States. The event began in May 2021 when wildlife rehabilitators and private citizens reported large numbers of sick and dead juvenile birds, mostly presenting as single cases with neurologic signs and/or ocular and periocular lesions. Early efforts by rehabilitators, veterinarians, state and federal wildlife agencies, and universities helped gather public reports and fuel rapid responses by government agencies. Collective efforts included live bird and carcass collections; submission to diagnostic laboratories and evaluation; information sharing; and coordinated messaging to stakeholders and interested parties. Extensive diagnostic evaluations failed to identify a causative pathogen or other etiology, although congruent results across laboratories have helped drive further investigation into alternative causes, such as nutritional deficiencies. This report highlights the strengths of a multi-agency, interdisciplinary investigation while exposing the need for an operational framework with approaches and resources dedicated to wildlife health.

## Simple Summary

In May 2021, wildlife managers in Washington D.C., Maryland, Virginia, West Virginia, and Kentucky began receiving reports of sick, dying, and dead birds with eye swelling and crusty discharge, some of which also exhibited neurological behaviors. The public and licensed wildlife rehabilitators provided initial reports, while additional birds were received in Delaware, New Jersey, Pennsylvania, Ohio, Indiana, Tennessee, and Connecticut. The majority of reports involved fledgling common grackles (*Quiscalus quiscula*), blue jays (*Cyanocitta cristata*), European starlings (*Sturnus vulgaris*), and American robins (*Turdus migratorius*). Early in the event, the jurisdictions involved indicated a collective desire to work together in a regional response with consistent public messaging and collaboration among diagnostic laboratories. The U.S. Geological Survey National Wildlife Health Center (NWHC) facilitated conversations regarding event response among the affected jurisdictions and coordinated with other responding diagnostic laboratories. However, despite exhaustive collective efforts, no definitive cause(s) of illness or death have been determined, with some analyses still ongoing. This paper provides additional background on the event, outlines approaches taken by agencies to coordinate their response and communications with the public, and summarizes lessons learned that could be used to inform future preparedness and response plans.

## 1. Introduction

Rapid detection and response to wildlife mortality events are key to understanding risks and minimizing potential population-level impacts and biodiversity loss [1,2]. Government agencies and private institutions often respond to mortality events with common goals of determining causative agent(s), identifying epidemiological patterns, assessing potential population impacts, and reducing potential risks to human and animal health [3]. However, the unpredictability of mortality events [4] coupled with the need for answers and the paucity of resources necessary to implement a unified response can impede investigations. Furthermore, such investigations often go undocumented particularly those of unknown etiology [5], making it difficult to draw from and build upon prior knowledge and experience in preparation for future responses to unknown threats [6].

We report on a rapid, coordinated response to a mortality event in multiple free-ranging passerine species in the eastern United States, with a focus on how lessons learned during the event could be used to promote successful collaborative response plans that facilitate management actions aimed at diminishing health threats to wildlife populations.

## 2. Morbidity and Mortality Event Background

**Figure 1:**
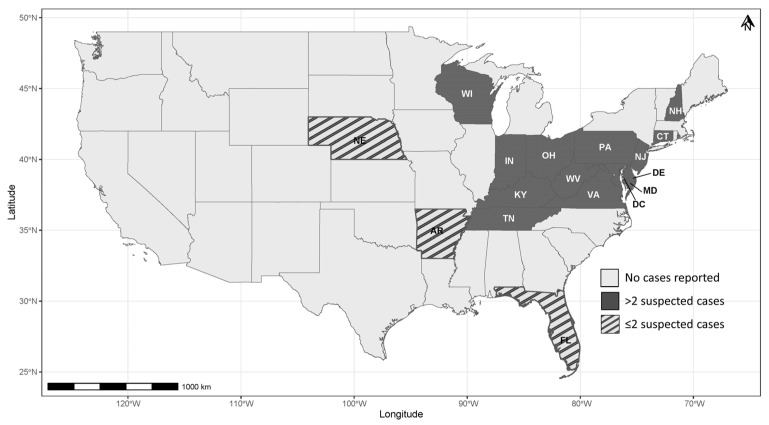
U.S. states in which suspect cases (i.e., those examined diagnostically and that had a history consistent with involvement and gross and/or microscopic evidence of blepharitis and/or conjunctivitis) were reported during the 2021 avian mortality event.

In May 2021, wildlife rehabilitators and private citizens began reporting large numbers of sick and dead juvenile (i.e., nestling and fledgling) passerines in areas of Maryland, Washington D.C., and Virginia [7]. Many reports included single juvenile birds with neurologic signs and/or ocular and periocular lesions found by the public and brought to wildlife rehabilitation facilities. Cases primarily involved three native species, the common grackle (*Quiscalus quiscula*), blue jay (*Cyanocitta cristata*), and American robin (*Turdus migratorius*), and one invasive species, the European starling (*Sturnus vulgaris*). In the following weeks, additional reports were received across the East Coast, including from West Virginia, Delaware, Connecticut, New Jersey, Pennsylvania, Ohio, Indiana, Kentucky, and Tennessee (Figure 1), with case numbers declining across all these states by mid-summer.

Early clinical observations included lethargy, loss of muscle control and coordination (i.e., ataxia), tremors, twisting of the neck and head (i.e., torticollis), rapid breathing (i.e., dyspnea), involuntary movements of the eye and eyelids (i.e., nystagmus and blepharospasm), corneal changes (i.e., edema, inflammation, and ulceration), conjunctival and eyelid swelling, ocular discharge, and poor response to visual stimuli. The involvement of soft tissues surrounding the eyes, in addition to the species affected and widespread geographic distribution, drew comparisons with those of Mycoplasma gallisepticum, first reported in wild house finches (*Haemorhous mexicanus*) in 1994 [8,9]. However, initial investigations by government agencies and academic partners did not reveal consistent detections of *Mycoplasma* spp. in affected birds, although *M. sturni* was detected in some samples. Furthermore, the neurologic signs exhibited by a large number of the birds have not previously been described in songbirds with mycoplasmosis.

The event also coincided temporally and spatially with the emergence of cicada brood X in 2021 [10]. Many bird nests belonging to both affected and unaffected birds were reported to be inundated with cicadas (*Magicicada* spp.), and many of the affected birds also had cicadas in their gastrointestinal tracts. The concurrence of the brood emergence with the observed bird mortalities prompted further investigations into the potential role of the cicadas and the associated anthropogenic activities, such as the use of insecticides or cicada-induced vitamin deficiency.

By late May, the apparently widespread geographical distribution of the event and its unknown etiology prompted state and federal wildlife agencies, veterinary diagnostic laboratories, universities, and research institutes to mount a collaborative response with local wildlife veterinarians and rehabilitators. The lack of causative or contributing etiologies to this event further fueled efforts to widen the discussion and number of experts involved to better evaluate other possible contributing causes: species biology; behavior; nutrition; toxins; caustic chemical exposure; and regional, seasonal, annual, or broader environmental changes. The immature ages of most suspected cases further indicated a possible role of developmental factors (e.g., limited mobility of non-flighted nestlings, incompletely developed immune systems, or yet unlearned defensive behaviors), while clinical disease in altricial nestlings may also have altered parental feeding and/or nurturing behaviors [11], potentially expediting health declines. Further, the role of commensal but potentially pathogenic or yet uncharacterized microorganisms, in the face of other stressors, cannot be ruled out, particularly considering disease development is often multifactorial and may be facilitated by seasonal or less predictable anthropogenic and/or environmental factors.

## 3. Summary of Diagnostic Findings

**Figure 2:**
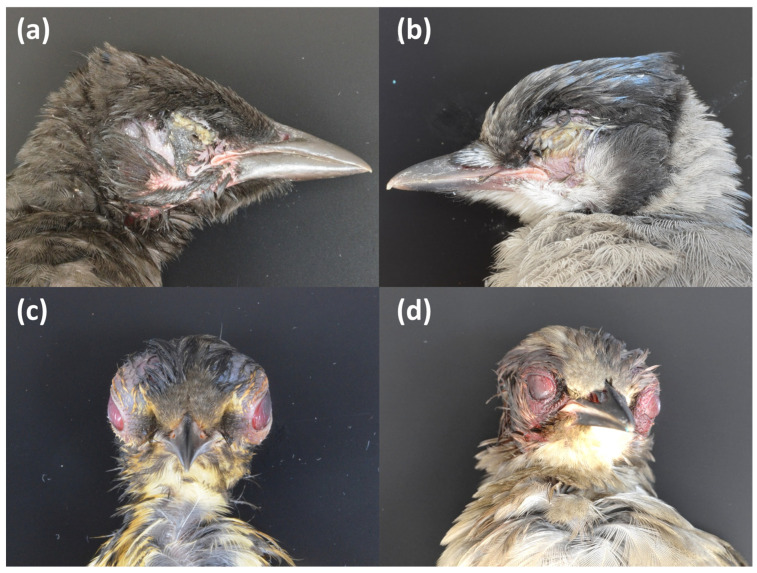
Characteristic gross lesions in passerines suspected to be involved in the event that were submitted to the Southeastern Cooperative Wildlife Disease Study (SCWDS) by the Virginia Department of Wildlife Resources. (**a**) Common grackle (*Quiscalus quiscula*) and (**b**) blue jay (*Cyanocitta cristata*) showing crusting ocular discharge. (**c**) American robin (*Turdus migratorius*) and (**d**) European starling (*Sturnus vulgaris*) showing bulging eyes with conjunctival swelling and periocular hemorrhage. Photo credit: SCWDS.

Whole carcasses and/or tissues of birds suspected to be involved in this morbidity and mortality event were submitted by state wildlife agencies, often in cooperation with wildlife rehabilitators and concerned members of the public, to veterinary and wildlife diagnostic laboratories (Table 1; [12]) for cross-disciplinary diagnostic evaluation including pathology, virology, microbiology, parasitology, and toxicology. When submitted birds were observed alive, clinical histories were provided and included those described above. The nutritional condition of subjects ranged from emaciated to good, with no discernible correlation to the severity of clinical observations. Gross pathology generally included bilateral swelling and erythema of the eyelids, often involving the conjunctiva and adjacent soft tissues, and/or ocular discharge and crusting. Some birds also had corneal ulceration, hyphema, and hemorrhage in periocular tissues and exophthalmos (Figure 2). Less often, overt evidence of traumatic injuries was also present, including skull fractures and internal hemorrhage (e.g., in the liver, kidneys, and lungs).

Histopathology confirmed that inflammation and edema of variable severity and distribution contributed to the eyelid and sometimes periocular tissue swelling (Figure 2), which would have been expected to greatly impair vision in many cases. Some cases also had mild to severe hemorrhage within these inflamed and edematous areas; while others occasionally had superficial (presumed opportunistic) bacterial invasion. Rarely, extension of inflammation from the periocular region to the brain and/or skull was observed. Inflammatory cell components were inconsistent but most often consisted of lymphocytes, plasma cells, and histiocytes, with varying proportions of heterophils. Some cases also had inflammation of the cornea and/or some internal ocular structures (i.e., iris, ciliary body, and uvea). Aside from these rare extensions of periocular inflammation to the brain, brain lesions were very rare, and thus it may be more likely that the appearance of neurologic-like signs in affected birds reflected the lack of coordination and other impacts associated with vision impairment from the periocular swelling and/or crusting, or secondary trauma in some cases.

Some cases had evidence of presumed secondary trauma, including skull fractures and periocular and intracelomic hemorrhage. Otherwise, most affected birds appeared in good general health with rare incidental findings such as small, random (not associated with the head), usually focal or few granulomas in various internal organs and renal or intestinal coccidiosis. This variability in clinical signs and nutritional condition could reflect the duration or chronicity of disease prior to death/euthanasia and circumstances of mortality (e.g., died in nest after failed parental care of short- or long-term duration due to lack of expected feeding responses; died from traumatic collision or fall from nest due to poor vision and/or morbidity; died or were euthanized after variable time periods in rehabilitation); however, with limited to no history on the evaluated birds, additional risk factors cannot be ruled out.

Pathogen-specific tests were performed across a number of diagnostic laboratories and research institutions and collectively were able to rule out many of the diseases and pathogens initially suspected, including *Mycoplasma* spp., *Chlamydia* spp., *Salmonella* spp., herpesviruses, West Nile virus, eastern equine encephalitis virus, avian paramyxoviruses, avian influenza viruses, adenoviruses, coronaviruses, poxviruses, and *Trichomonas* spp. Non-targeted toxicology testing of liver tissue, namely gas and liquid chromatography–mass spectrometry [GC/LC-MS] and mineral/heavy metal screening [ICP-MS], detected organochlorine insecticide residues among some birds. However, these detections were attributed to chronic environmental exposures and were not considered to have contributed to acute morbidity and mortality based on the very low concentrations, lack of consistent detections among the birds tested, and holistic antemortem and postmortem findings. High levels of iron were reported in some of the affected birds, although it remains unclear if this was a secondary effect with high iron levels often seen secondary to starvation in birds [13]. Several cicadas also were tested for heavy metals and salts and via LC-MS and GC-MS for other major organic compounds including cathinone and psilocybin, which are behavior-modifying chemicals produced by a fungus that commonly infect cicadas but have unknown effects on species that consume the infected insects [14]. No organic compounds were detected, while several salts (calcium, magnesium, phosphorus, potassium, sodium, and sulfur) and heavy metals (copper, iron, manganese, molybdenum, and zinc) were consistently detected, although the significance of these remains poorly understood.

The search for the cause of this mortality event further inspired collaborative efforts using metagenomic next-generation sequencing (mNGS, [15]). This effort involved three laboratories that independently received and tested samples for mNGS utilizing different experimental designs before working together on parallel analyses to help increase confidence in the findings. Collectively, several bacterial pathogens were detected significantly more often in the affected birds when compared to unaffected controls, including *Mycoplasma* spp. and *Avibacterium* spp.; however, these were deemed unlikely drivers of the mortality event and highlight the importance of having baseline data for comparison between diseased and healthy individuals when responding to future wildlife mortality events [15].

## 4. Communication and Coordination

**Figure 3:**
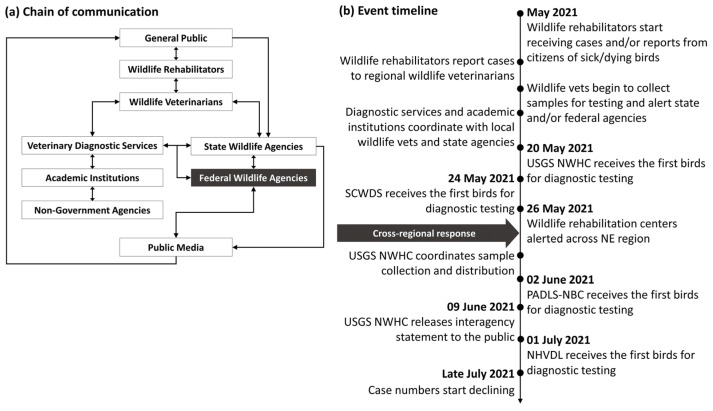
Alignment between (**a**) the communication chain starting with private citizens reporting sick or dead birds to wildlife rehabilitators and (**b**) the event timeline for the 2021 songbird mortality event. PADLS-NBC: Pennsylvania Animal Diagnostic Laboratory System—New Bolton Center; SCWDS: Southeastern Cooperative Wildlife Disease Study; USGS NWHC: United States Geological Survey National Wildlife Health Center; NHVDL: New Hampshire Veterinary Diagnostic Laboratory.

As initial cases started to build, communication among interested parties relied heavily on personal contacts (i.e., messages and/or phone calls between wildlife rehabilitators and veterinarians). Figure 3 depicts how the communication network among different stakeholders unfolded over the course of the event. Early in the response, delays in communication were seen as formal reporting processes (i.e., what, when, and whom to report to) and were not always clear. If non-agency veterinarians and wildlife rehabilitators had established agency contacts (with an onus on the agency—information campaign, social media, website, etc.), and agencies and veterinarians had diagnostic laboratory contacts already within arm’s reach with ideally established relationships, it may have been feasible to launch a coordinated national response earlier. A rapid response is particularly important in short-lived events such as this; for instance, by the time national calls began and efforts to coordinate testing were underway, case numbers were already declining. As the event progressed into June, the U.S. Geological Survey (USGS) National Wildlife Health Center started hosting virtual meetings among involved and interested parties, primarily from state and federal agencies and universities. Discussions promoted consistent public messaging and engagement in group discussions to establish consistent field and diagnostic approaches. Collaboratively, hundreds of birds were field-collected with the assistance of wildlife rehabilitators, veterinary clinics, concerned citizens, and state wildlife agencies. Early communications among local agencies, rehabilitators, veterinarians, diagnostic laboratories, and the federal government helped channel public awareness and manage expectations when reporting sick or dead birds, with some agencies utilizing telephone hotlines or online data forms to record public observations. The latter method facilitated information gathering and sample collection by agencies but may also have inspired increased reporting by the public, which included reports of birds being struck by vehicles, bird–window strikes, infectious diseases (e.g., avian pox, salmonellosis), and other forms of bird morbidity and mortality that were likely not epidemiologically linked and made defining the true scale of the event more difficult.

Initial attempts to establish a case definition or criteria reflected the early clinical observations involving conjunctival and eyelid swelling and ocular discharge. The species, age, and location of affected birds were further considered in the definition with updates being made based on the available information. For instance, early in the event the distribution of cases was limited to the East Central (mid-Atlantic) U.S. region. However, it soon expanded to states in the Northeast and Western Great Lakes regions, with few birds being reported from outside these regions (Figure 1). These seemingly disjointed regions suspected of being involved in the event, in addition to suspicions of additional, varied species (e.g., non-passerines such as raptors) being involved and inconsistent clinical signs and gross presentation, collectively made it difficult to hone in on an accurate case definition. Consequently, it was challenging to define and track the scope of the morbidity and mortality event. For example, northern cardinals (*Cardinalis cardinalis*) suspected of being involved were sampled in high numbers in both Maryland and Delaware with only a single sample outside of these two states (Table 1). However, in other states, northern cardinals were reported by the public in high numbers, such as in Pennsylvania where they received 173 online reports of cardinals over the course of the event. However, of these reports, only ten were juvenile birds, among which four had reported eye abnormalities, resulting in a large uncertainty as to whether these birds were true cases. Further, the lack of identification of the cause of morbidity precluded the ability to diagnostically confirm any of the suspect cases. This, coupled with a lack of comprehensive epidemiological investigations, such as proactive (versus passive) surveillance in further defining the affected areas, species, and potential involvement of environmental factors (e.g., weather conditions, availability of optimal food and habitat, and presence of insect vectors), impeded the understanding of the event, much less how to message it to the concerned public, reveal possible risks to human, animal, and environmental health, and consider disease management strategies.

## 5. Challenges and Future Directions

The multifactorial and complex nature of wildlife mortality events is driven by dynamic relationships among the host, environment, etiologic agent (e.g., a pathogen or toxin), and sometimes human and domestic animal activities. These events also are exacerbated by underlying stressors on wildlife, such as limited resource availability and/or quality, and accelerated rates of global climate change [16]. Thus, establishing the cause(s) and associations of wildlife mortality events, especially those that are unforeseen or unusual, in a timely fashion, is challenging. Moreover, challenges to understanding these events go well beyond science alone. Our collaborative approach to this songbird mortality event response highlighted other major limitations. First, the limited availability of resources and personnel for wildlife investigations compared to those of human and/or livestock poses serious constraints to our ability to respond efficiently and effectively to wildlife disease outbreaks [17,18]. Further constraints can arise due to the distinct missions, goals, and approaches among local, state, and federal regulatory agencies, which can challenge inter-agency communication, jurisdictional responsibilities, and coordination in the use of resources, funding, and information sharing [19]. These limitations influence not only response activities but also broader wildlife health surveillance programs. An operational framework, in the context of One Health, that acknowledges the shared risks between animals and humans, would be useful to strengthen coordinated responses for wildlife health. Such a framework could have been used during this morbidity and mortality event to help define networks of partners with different areas of expertise both at regional and national scales and accelerate integrative efforts towards synergistic and standardized protocols without the need for regulatory mandates. Such efforts help not only with long-term strategic planning but also with the allocation of resources and ensuring adequate training and preparation across stakeholder groups.

This response further highlighted the importance of coordinated public messaging. Throughout the event, the public demonstrated concern about wild passerine morbidity and mortality, which both facilitated sample collection and stimulated media interest. Online public reporting forms generated by state wildlife agencies facilitated rapid information gathering and sample collection but also increased reporting by the public of traumatic injuries and other causes of morbidity/mortality, making identifying the true scale of the event difficult. Early in the event, messaging discrepancies surfaced among government agencies, such as general guidance on removing bird feeders during the event, and potentially increased the public’s concerns and questions. The success of the robust public involvement through reporting observations and sample collections, however, fuels the aforementioned need for available resources and existing communication frameworks among wildlife agencies and interested partners. One consideration to reduce the burden across partners would be to have a pre-designated coordinator for future events who would be responsible for gathering and sharing information across the response network and summarizing findings in a coordinated manner to inform public messaging.

The substantial effect of social and political factors and human behavior on management during a mortality event was evident almost immediately. The concurrent COVID-19 pandemic with shelter-in-place protocols increased the public’s desire to observe and connect with nature [20]. This phenomenon likely influenced public perception, enhanced interest in this event, and further stimulated media response. It also highlighted the importance of increasing public awareness of the benefits provided by wildlife and intact, healthy ecosystems (e.g., recreation, education, clean air and water, nutrient-filled soils, pollination, medicines, and climate) [21]. The public interest also generated a need for answers early on in the event, including the impact of human behavior, such as chemical spraying to control cicadas, as a trigger for the unusual mortality event; this further highlights the benefit of coordinated communication with the public across agencies. Enhancing communication networks among wildlife health partners is just one component of effective emergency response plans that may be used to help prepare for future wildlife health events [22,23,24].

## 6. Conclusions

The response to the 2021 songbird mortality event in the eastern United States highlighted the importance of adequate resources dedicated to wildlife, as well as the integration of wildlife into operational frameworks at regional and national scales. The expertise needed in such events is broad and varied, and it may include wildlife health, wildlife biology, epidemiology, veterinary medicine, pathology, microbiology, toxicology, genetics, environmental science, natural resource management, public health, climate science, and other disciplines. Such frameworks would help provide a unified set of guidelines and promote timely, adaptive, and collaborative responses to wildlife health events at the regional, state, and national levels, facilitating a more consistent, robust, and effective approach to wildlife health and management.

## Figures and Tables

**Table 1:** The number of suspect cases (i.e., those examined diagnostically and that had a history consistent with involvement and gross and/or microscopic evidence of blepharitis and/or conjunctivitis) reported from May to September 2021 and submitted for diagnostic evaluation by species, age, sex, origin state (county in the footnotes), and date. PADLS-NBC: Pennsylvania Animal Diagnostic Laboratory System—New Bolton Center; SCWDS: Southeastern Cooperative Wildlife Disease Study; NWHC: USGS National Wildlife Health Center; NHVDL: New Hampshire Veterinary Diagnostic Laboratory.

Species Common Name (Scientific Name)	Diagnostic Lab				Total
	PADLS-NBC	SCWDS	NWHC	NHVDL	
American Robin (*Turdus migratorius*)	6	4	1	15	26
Blue Jay (*Cyanocitta cristata*)	102	27	13	31	173
Common Grackle (*Quiscalus quiscula*)	23	8	6	13	50
Cooper’s Hawk (*Accipiter cooperii*)	-	-	-	5	5
Crow (*Corvus* sp.)	-	-	-	1	1
European Starling (*Sturnus vulgaris*)	4	15	2	11	32
Eastern Phoebe (*Sayornis phoebe*)	-	-	-	1	1
Eastern Screech Owl (*Megascops asio*)	-	-	-	1	1
Mourning Dove (*Zenaida macroura*)	-	-	-	2	2
Northern Mockingbird (*Mimus polyglottos*)	2	-	-	-	2
Northern Cardinal (*Cardinalis cardinalis*)	12	-	-	1	13
House Sparrow (*Passer domesticus*)	1	-	-	5	6
Finch (*Fringillidae* spp.)	-	-	-	6	6
Pigeon (*Columbidae* sp.)	-	-	-	1	1
Rose-breasted Grosbeak (*Pheucticus ludovicianus*)	-	-	-	1	1
Rusty Blackbird (*Euphagus carolinus*)	-	-	-	1	1
Sharp-shinned Hawk (*Accipiter striatus*)	-	-	-	1	1
Thrush (*Turdus spp.*)	-	-	-	2	2
Tufted Titmouse (*Baeolophus bicolor*)	-	-	-	1	1
**Age**					
Adult	1	-	-	-	1
Juvenile (nestlings/fledglings)	149	57	22	-	228
Unknown	-	-	-	99	99
**Sex**					
Male	-	10	5	-	15
Female	-	7	6	-	13
Unknown	150	37	11	99	297
State *					
MD	128	-	10	13	151
PA	20	-	-	-	20
DE	2	-	-	-	2
KY	-	19	-	-	19
VA	-	27	-	2	29
WV	-	6	-	-	6
AR	-	1	-	-	1
FL	-	1	2		3
D.C.	-	-	4	20	24
OH	-	-	4	5	9
WI	-	-	1	1	2
NE	-	-	1	0	1
CT	-	-	-	29	29
NH	-	-	-	6	6
NJ	-	-	-	23	23
**Date found**					
First case	10 May 2021	24 May 2021	20 May 2021	14 May 2021	-
Last case	10 September 2021	27 August 2021	22 August 2021	21 September 2021	-

* Counties by state: VA: Frederick, Fairfax, Arlington, Loudoun, Clarke, Berkeley, Shenandoah; WV: Berkeley, Jefferson; KY: Jefferson, Boone, Campbell, Kenton, Grant; FL: Broward, Lee; AR: Faulkner; NE: Lancaster; OH: Delaware, Franklin, Hamilton, Montgomery; PA: Allegheny, Chester, Crawford, Cumberland, Dauphin, Huntingdon, Lancaster, Monroe, Montgomery, Northampton, Philadelphia, Warren, Wyoming, York; MD: Anne Arundel, Carroll, Cecil, Frederick, Howard, Montgomery, Prince George’s; DE: New Castle; WI: Pierce.

## Data Availability

No new data were created or analyzed in this study. Data sharing does not apply to this article.

## References

[1] Grogan L.F., Berger L., Rose K., Grillo V., Cashins S.D., Skerratt L.F. (2014). Surveillance for emerging biodiversity diseases of wildlife. PLoS Pathog..

[2] Kelly T.R., Pandit P.S., Carion N., Dombrowski D.F., Rogers K.H., McMillin S.C., Clifford D.L., Riberi A., Ziccardi M.H., Donnelly-Greenan E.L. (2021). Early detection of wildlife morbidity and mortality through an event-based surveillance system. Proc. R. Soc. B Biol. Sci..

[3] Ryser-Degiorgis M.-P. (2013). Wildlife health investigations: Needs, challenges and recommendations. BMC Vet. Res..

[4] Fey S.B., Siepielski A.M., Nusslé S., Cervantes-Yoshida K., Hwan J.L., Huber E.R., Fey M.J., Catenazzi A., Carlson S.M. (2015). Recent shifts in the occurrence, cause, and magnitude of animal mass mortality events. Proc. Natl. Acad. Sci. USA.

[5] Delgado M., Ferrari N., Fanelli A., Muset S., Thompson L., Sleeman J.M., White C.L., Walsh D., Wannous C., Tizzani P. (2023). Wildlife health surveillance: Gaps, needs and opportunities. Rev. Sci. Tech. Off. Int. Epizoot..

[6] Morner T., Obendorf D.L., Artois M., Woodford M.H. (2002). Surveillance and monitoring of wildlife diseases. Rev. Sci. Tech. Off. Int. Epiz..

[7] USGS Interagency Statement: USGS and Partners Investigating DC Area Bird Mortality Event. https://www.usgs.gov/news/national-news-release/interagency-statement-usgs-and-partners-investigating-dc-area-bird.

[8] Fischer J.R., Stallknecht D.E., Luttrell P., Dhondt A.A., Converse K.A. (1997). Mycoplasmal conjunctivitis in wild songbirds: The spread of a new contagious disease in a mobile host population. Emerg. Infect. Dis..

[9] Ley D.H., Berkhoff J.E., McLaren J.M. (1996). Mycoplasma gallisepticum Isolated from House Finches (Carpodacus Mexicanus) with Conjunctivitis. Avian Dis..

[10] Kritsky G. (2021). One for the books: The 2021 emergence of the periodical cicada brood X. Am. Entomol..

[11] Klug H., Bonsall M.B. (2014). What are the benefits of parental care? The importance of parental effects on developmental rate. Ecol. Evol..

[12] Grear D.A., Lorch J.M. (2024). Data Release: Coordinated Efforts Toward Understanding a Mortality Event in Wild Passerines in the Eastern United States: Responses, Lessons Learned and Future Recommendations.

[13] Cork S.C. (2000). Iron storage diseases in birds. Avian Pathol..

[14] Boyce G.R., Gluck-Thaler E., Slot J.C., Stajich J.E., Davis W.J., James T.Y., Cooley J.R., Panaccione D.G., Eilenberg J., De Fine Licht H.H. (2019). Psychoactive plant- and mushroom-associated alkaloids from two behavior modifying cicada pathogens. Fungal Ecol..

[15] Mwakibete L., Greening S.S., Kalantar K., Ahyong V., Anis E., Miller E.A., Needle D.B., Oglesbee M., Thomas W.K., Sevigny J.L. (2024). Metagenomics for pathogen detection during a mass mortality event in songbirds. J. Wildl. Dis..

[16] Acevedo-Whitehouse K., Duffus A.L.J. (2009). Effects of environmental change on wildlife health. Philos. Trans. R. Soc. B Biol. Sci..

[17] World Bank Group (2012). People, Pathogens and Our Planet: The Economics of One Health.

[18] Machalaba C., Uhart M., Ryser-Degiorgis M.-P., Karesh W.B. (2021). Gaps in health security related to wildlife and environment affecting pandemic prevention and preparedness, 2007–2020. Bull. World Health Organ..

[19] Willette M., Schott R. (2017). Response planning for wildlife rehabilitation centers: An infectious disease management policy—Highly pathogenic avian influenza. Wildl. Rehabil. Bull..

[20] Schwartz M.W., Glikman J.A., Cook C.N. (2020). The COVID-19 Pandemic: A learnable moment for conservation. Conserv. Sci. Pract..

[21] Daily G.C., Polasky S., Goldstein J., Kareiva P.M., Mooney H.A., Pejchar L., Ricketts T.H., Salzman J., Shallenberger R. (2009). Ecosystem services in decision making: Time to deliver. Front. Ecol. Environ..

[22] Wildlife Health Australia (2023). Guidelines for Management of an Emergency Wildlife Disease Response.

[23] ECCC (2020). Guidelines for Effective Wildlife Response Plans.

[24] Gravem S., Bachhuber S., Burnaford J., Field L., Gavenus K., Groner M., Hamilton S., Jaffe N., Schiebelhut L., Miner M. (2023). A Research Network and Contingency Plan for Monitoring and Responding to Marine Disease Emergencies.

